# Ablation of Sim1 Neurons Causes Obesity through Hyperphagia and Reduced Energy Expenditure

**DOI:** 10.1371/journal.pone.0036453

**Published:** 2012-04-27

**Authors:** Dong Xi, Nilay Gandhi, Meizan Lai, Bassil M. Kublaoui

**Affiliations:** Division of Endocrinology and Diabetes, Department of Pediatrics, School of Medicine, The Children's Hospital of Philadelphia, University of Pennsylvania, Philadelphia, Pennsylvania, United States of America; University of Cordoba, Spain

## Abstract

Single-minded 1 (Sim1) is a transcription factor necessary for development of the paraventricular nucleus of the hypothalamus (PVH). This nucleus is a critical regulator of appetite, energy expenditure and body weight. Previously we showed that *Sim1^+/−^* mice and conditional postnatal *Sim1^−/−^* mice exhibit hyperphagia, obesity, increased linear growth and susceptibility to diet-induced obesity, but no decrease in energy expenditure. Bilateral ablation of the PVH causes obesity due to hyperphagia and reduced energy expenditure. It remains unknown whether Sim1 neurons regulate energy expenditure. In this study, Sim1cre mice were bred to homozygous inducible diphtheria toxin receptor (iDTR) mice to generate mice expressing the simian DTR in Sim1 cells. In these mice, Sim1 neuron ablation was performed by intracerebroventricular (ICV) injection of diphtheria toxin. Compared to controls, mice with Sim1 neuron ablation became obese (with increased fat mass) on a chow diet due to increased food intake and reduced energy expenditure. In post-injection mice, we observed a strong inverse correlation between the degree of obesity and hypothalamic *Sim1* expression. The reduction in baseline energy expenditure observed in these mice was accompanied by a reduction in activity. This reduction in activity did not fully account for the reduced energy expenditure as these mice exhibited decreased resting energy expenditure, decreased body temperature, decreased brown adipose tissue temperature, and decreased UCP1 expression suggesting an impairment of thermogenesis. In injected mice, hypothalamic gene expression of *Sim1*, *oxytocin* (*OXT*) and *thyrotropin releasing hormone* (*TRH*) was reduced by about 50%. These results demonstrate that Sim1 neurons in adult mice regulate both food intake and energy expenditure. Based on the body of work in the field, feeding regulation by Sim1 neurons likely occurs in both the PVH and medial amygdala, in contrast to energy expenditure regulation by Sim1 neurons, which likely is localized to the PVH.

## Introduction

Homeostatic regulation of feeding and energy expenditure is distributed among many brain regions [1], [2] with the paraventricular nucleus of the hypothalamus (PVH) playing an important role. The PVH receives projections from leptin responsive neurons in the arcuate nucleus [3] and has a massive projection to the solitary nucleus (NTS) [4], [5] and the sympathetic intermediolateral column of the spinal cord (IML) [6], [7]. Both hypophysiotropic and descending parvocellular divisions of the PVH are activated by leptin [8], [9]. Contralateral knife cuts severing PVH output on one side and NTS input on the other lead to hyperphagic obesity [10]. Injection of melanocortin agonists into the PVH reduces food intake (FI) while injection of melanocortin antagonists and neuropeptide Y (NPY) increases FI [11]. The PVH is widely accepted to have a role in sympathetic outflow to brown adipose tissue (BAT) [12], [13] in addition to projections to the preoptic area (POA) and the dorsomedial hypothalamic nucleus (DMH) [14], [15] regions critical for regulation of thermogenesis [16]. Pseudorabies virus (PRV) transneuronal labeling experiments have established a clear link between BAT and the PVH [17], [18]. NPY injection into the PVH also suppresses sympathetic nerve activity to interscapular brown adipose tissue [19]. Moreover, the PVH (specifically the dorsal parvocellular and ventromedial parvocellular divisions) is a major source of descending projections to sympathetic preganglionic neurons of the IML of the spinal cord [7]. The above studies implicate the PVH in both FI and energy expenditure (EE) regulation.

Single-minded 1 (Sim1) is expressed in the PVH, supraoptic nucleus (SON), medial amygdala and nucleus of the lateral olfactory tract (NLOT) [20], [21]. Our work with *Sim1^+/−^* mice and conditional postnatal *Sim1^−/−^* mice revealed that they are hyperphagic and obese, but exhibit normal EE [22]–[24]. Overexpression of Sim1 partially rescues agouti yellow obesity by normalizing food intake without altering feeding efficiency, a marker of energy expenditure [25]. Knockdown of Sim1 in mouse PVH leads to increased food intake and overexpression to reduced food intake [26], and postnatal Sim1 deficiency causes hyperphagic obesity [24], confirming a physiologic role for Sim1 beyond development. Sim1 deficiency leads to a marked reduction in melanocortin 4 receptor (MC4R) expression in the PVH [24] and resistance to c-FOS activation of PVH neurons by the melanocortin agonist melanotan II (MTII) [23]. This is associated with resistance to MTII's anorectic effect but not MTII's effect on energy expenditure [23]. This result suggests that Sim1 acts as a transcription factor via melanocortin receptors in the PVH to regulate feeding, but has no role in energy expenditure regulation. This hypothesis is supported by the work of Elmquist and colleagues who showed that reactivation of MC4R expression (in MC4R null mice) in Sim1 neurons rescues food intake with no effect on energy expenditure [20], consistent with the idea that MC4Rs in the PVH regulate food intake while MC4Rs elsewhere regulate energy expenditure.

These results were surprising in light of the fact that Sim1 neurons account for most if not all neurons of the PVH [21] and bilateral electrolytic ablation of the PVH leads to reduced energy expenditure, and lack of diet induced thermogenesis [27], [28] in addition to increased food intake, and obesity [27]–[29]. In addition, the injection of melanocortin agonists into the PVH increases energy expenditure [11]. The dispensability of the Sim1 gene in regulating energy expenditure does not mean that Sim1 neurons do not play a role in energy expenditure regulation. We know from other knockout models that neurons can function normally even when their signature gene or neuropeptide is knocked out, making ablation of specific neurons desirable. One example of this phenomenon is seen in mice with a double knockout of *NPY* and agouti related peptide (*AgRP*); these mice displayed no feeding phenotype [30], whereas ablation of NPY/AgRP neurons led to starvation within days [31], [32]. While it appears that the Sim1 gene has no ongoing role in energy expenditure regulation it is unclear if Sim1 neurons have such a role. Here we use the inducible diphtheria toxin receptor (iDTR) model [33], [34] to partially ablate Sim1 neurons in adult mice and examine the effect on feeding, energy expenditure regulation, activity, thermogenesis, and body weight.

## Results

### SIM1 neuron ablation in adult mice

**Figure 1:**
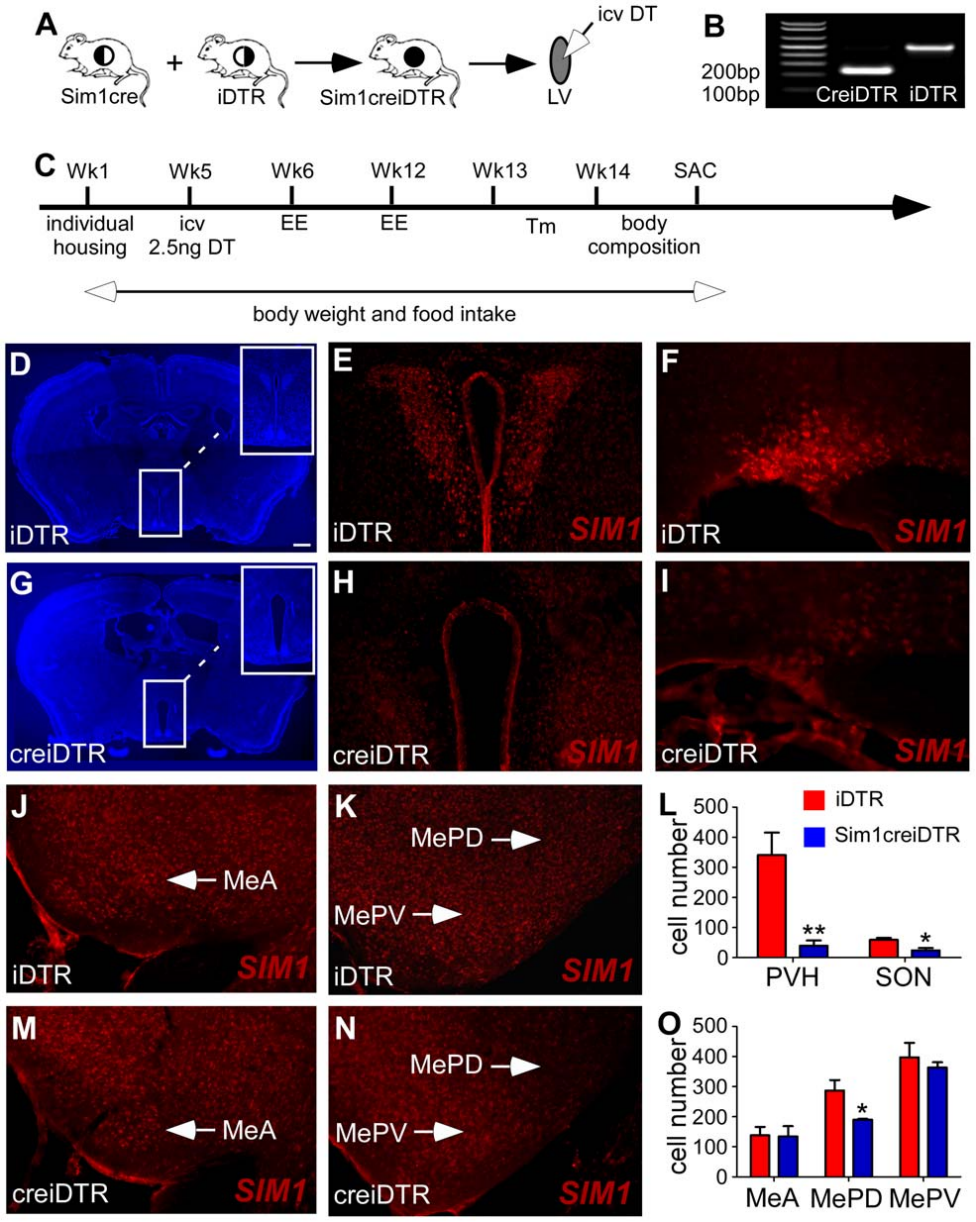
Generation of Sim1creiDTR mice and experimental schedule. (A) Sim1cre mice were crossed with homozygous iDTR mice to generate Sim1creiDTR mice and iDTR littermates. (B) Mouse genotyping shows that the cre gene product of 250 bp is expressed in Sim1creiDTR mice, while a normal product of 550 bp is amplified from iDTR mice that do not express cre. (C) Detailed experimental schedule. 6-week-old female mice were entered into the study (Wk1). Cannulation and DT injection took place at Wk5. Body weight and food intake were measured weekly before and after injection of DT. Energy expenditure was measured at wk 6 and wk 12. Body and brown adipose tissue temperature and body composition were measured before the mice were sacrificed. (D) Coronal sections of iDTR mouse brain stained with DAPI showing a dense PVH, with decreased density in Sim1creiDTR mice (G). Immunofluorescence staining with an antibody to SIM1 in PVH (E, H), SON (F, I), MeA (J, M), MePD and MePV (K, N). Quantitation of cell number in each region (L, O, n = 3 for each group, *p<0.05; **p<0.01). Scale bar: 200 µm for D, G; 40 µm for E, H, J, K, M, N; and 20 µm for F, I.

To investigate the role of SIM1 neurons in regulating energy balance in adult mice, we generated Sim1creiDTR mice by crossing heterozygous Sim1cre mice with homozygous iDTR mice (Fig. 1A). Sim1creiDTR mice express the simian diphtheria toxin receptor in SIM1 neurons making them susceptible to diphtheria toxin mediated ablation. Genotyping of offspring (Fig. 1B) revealed the expected size of the amplified product in Sim1creiDTR mice, vs. cre-negative iDTR littermates. Figure 1C shows a detailed experimental schedule. 6-week-old female mice of both genotypes were habituated to individual housing (Wk1), then implanted with an ICV cannula in the lateral ventricle at Wk5 (Fig. 1C) and allowed to recover. All mice were injected with 2.5ng of diphtheria toxin (DT) followed by measurement of food intake, body weight, and energy expenditure, as outlined. Figure 1D, G shows DAPI staining of representative mouse PVH showing loss of cells in Sim1creiDTR mice compared to iDTR mice treated with DT. Representative images of SIM1 immunostaining (Fig. 1 E, F, H, I, J, K, M, N) show a decrease in the number of SIM1-positive cells in PVH (−88%, Fig. 1L, p<0.01, df = 2), SON (−59%, Fig. 1L, p<0.05, dF = 3) and the posterodorsal part of the medial amygadloid nucleus (MePD) (−34%, Fig. 1O, p<0.05, df = 2) in Sim1creiDTR mice, but not in the anterior part of the medial amygdaloid nucleus (MeA) or the posteroventral part of the medial amygadloid nucleus (MePV) (Fig. 1O).

### SIM1 neuron ablation causes hyperphagia and profound obesity

**Figure 2:**
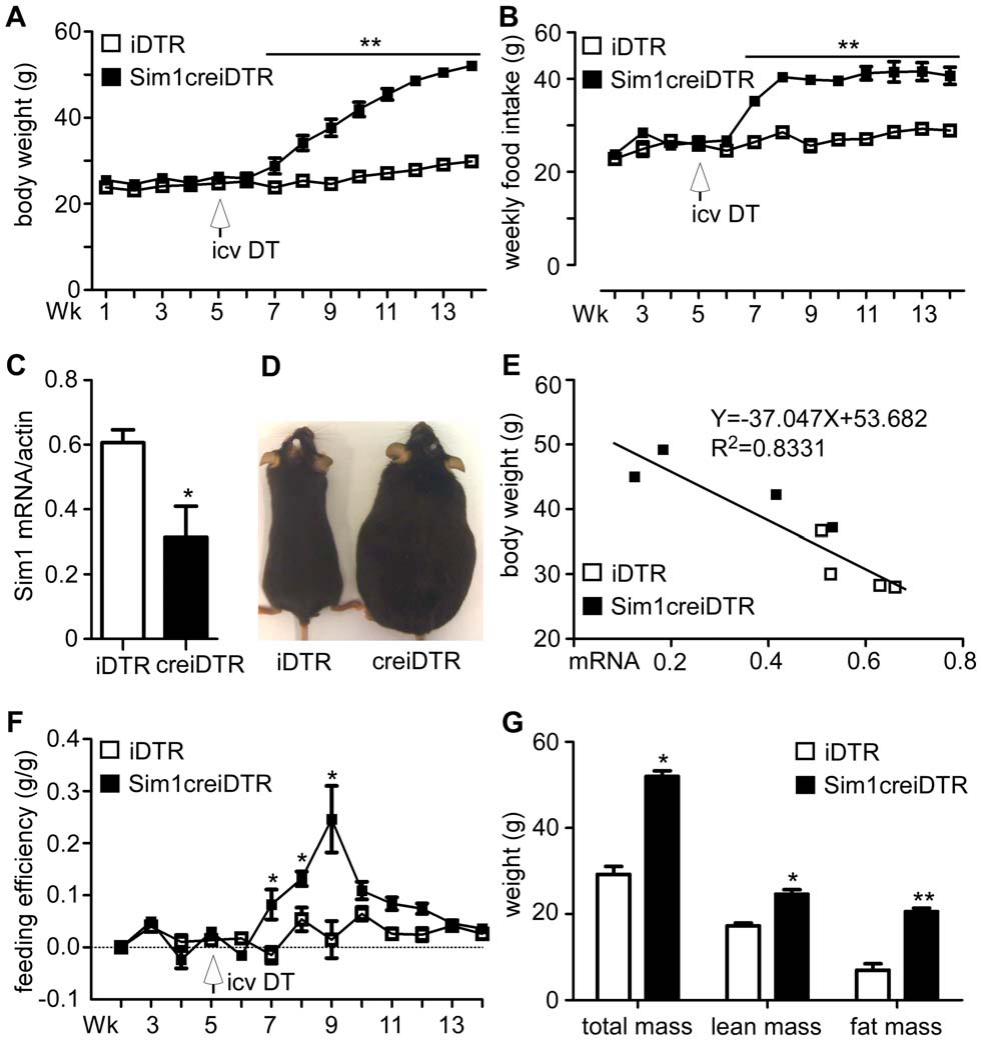
Ablation of Sim1 neurons causes hyperphagic obesity. (A) Growth curves and (B) weekly food intake of Sim1creiDTR versus iDTR mice. 2.5 ng DT was ICV injected at wk 5. Body weight and food intake were measured weekly on a chow diet (n = 7 for each group, *p<0.05). (C) Hypothalamic mRNA expression of Sim1creiDTR mice versus iDTR mice (n = 4 for each group, *p<0.05). (D) Representative images of iDTR and Sim1creiDTR mice (wk 14). (E) Relationship between final body weight and hypothalamic *Sim1* mRNA levels of Sim1creiDTR mice and iDTR mice. (F) Feeding efficiency calculated as the ratio between weekly body weight change (g) and food intake (g) (n = 7 for each group, *p<0.05). (G) Body composition measured by NMR (n = 4 for each group, *p<0.05; **p<0.01). Error bars indicate SEM for all figures. Means at each time point or condition were compared by a two-tailed *t*-test, with Welch's correction.

Sim1 expressing neurons in the PVH account for a majority if not all neurons in the PVH [21]. Sim1 has been shown to have both a developmental as well as a postdevelopmental physiologic role in feeding regulation [21], [23], [24], [26], [35]. To further explore whether SIM1 neurons regulate both food intake and energy expenditure, we examined body weight and food intake. Prior to DT treatment, iDTR and Sim1creiDTR mice showed no difference in body weight (Fig. 2A, wk 1–5) or weekly food intake (Fig. 2B, wk 2–5) on a chow diet (p>0.05). All mice were ICV injected with DT on wk 5 of the protocol. Body weight between ablated mice and non-ablated mice began to diverge 2 weeks after injection (wk 7). Final body weight of DT treated Sim1creiDTR mice (52.06±0.93 g) was 1.7 times greater than DT treated iDTR littermates (29.86±1.19 g) (p<0.05, df = 11). The increase in body weight correlated with a concurrent increase in food intake (Fig. 2B) starting 2 weeks after DT treatment (p<0.05, df = 7). Food intake reached a plateau at 40.69±1.85 g/wk (152.59±6.94 kcal/wk) 3 weeks after DT treatment (wk 8), which is 1.4-fold greater than the intake of iDTR mice (28.90±0.30 g/wk or 108.38±1.13 kcal/wk) (p<0.01, df = 6). Compared to iDTR mice, Sim1creiDTR mice ate more during both light and dark cycles with a greater difference in the light cycle (+98%) vs. the dark cycle (+35%, data no shown). Expression of *Sim1* mRNA in hypothalami of Sim1creiDTR mice was significantly decreased (Fig. 2C), 0.31±0.10 vs. 0.61±0.04 for iDTR mice confirming the successful partial ablation of SIM1 neurons. An image of representative mice from both groups is consistent with Sim1creiDTR mice being significantly overweight (Fig. 2D). Figure 2E shows body weight vs. hypothalamic mRNA expression of *Sim1*for individual mice from both groups. There was a significant negative correlation between body weight and hypothalamic *Sim1* expression (R^2^ = 0.8331), confirming that mice with more extensive loss of *Sim1* expression developed more severe obesity. Feeding efficiency (Fig. 2F) of Sim1creiDTR and iDTR mice overlapped before injection of DT, and began to diverge 2 weeks after injection. Sim1creiDTR mice exhibited an increase in feeding efficiency at wk 7, peaking at wk 9, and returning to normal between wk 10 and 13. To further explore the effects of adult Sim1 neuron ablation on metabolism, we measured body composition by nuclear magnetic resonance (NMR). In DT treated Sim1creiDTR mice compared to iDTR mice, total body mass was increased by 1.8 fold, lean mass by 1.4 fold (Fig. 2G, p<0.05, df = 5), and fat mass by 2.9 fold (20.59±0.79 g vs. 6.99±1.49 g, p<0.01, df = 4). Furthermore, the weight of epigonadal fat pads was significantly increased in Sim1creiDTR mice by 3.3 fold (data not shown, p<0.01, df = 5). Male mice exhibited a similar trend in weight gain but were less severely affected (25% vs. 59% increase in body weight, 6 weeks after DT injection).

### SIM1 neuron ablation reduces energy expenditure and activity and disrupts thermogenesis

**Figure 3:**
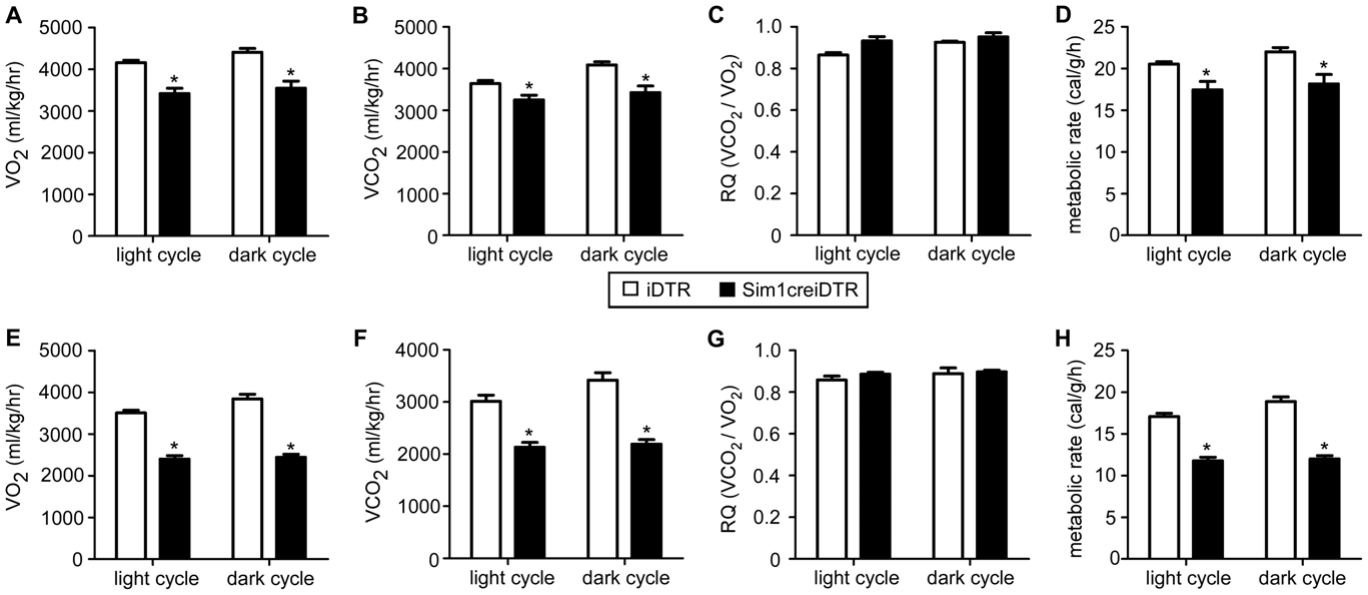
Ablation of Sim1 neurons reduces energy expenditure. Energy expenditure measured in CLAMS cages over a 72-hr period in the pre-obese state at wk 6 (A–D) and in the obese state wk 12 (E–H). Measures during light and dark cycles are presented separately. VO_2_, VCO_2_ and metabolic rate were normalized to body weight (n = 4 for each group, *p<0.05). Error bars indicate SEM for all figures. Means for each condition were compared by a two-tailed *t*-test, with Welch's correction.

**Figure 4:**
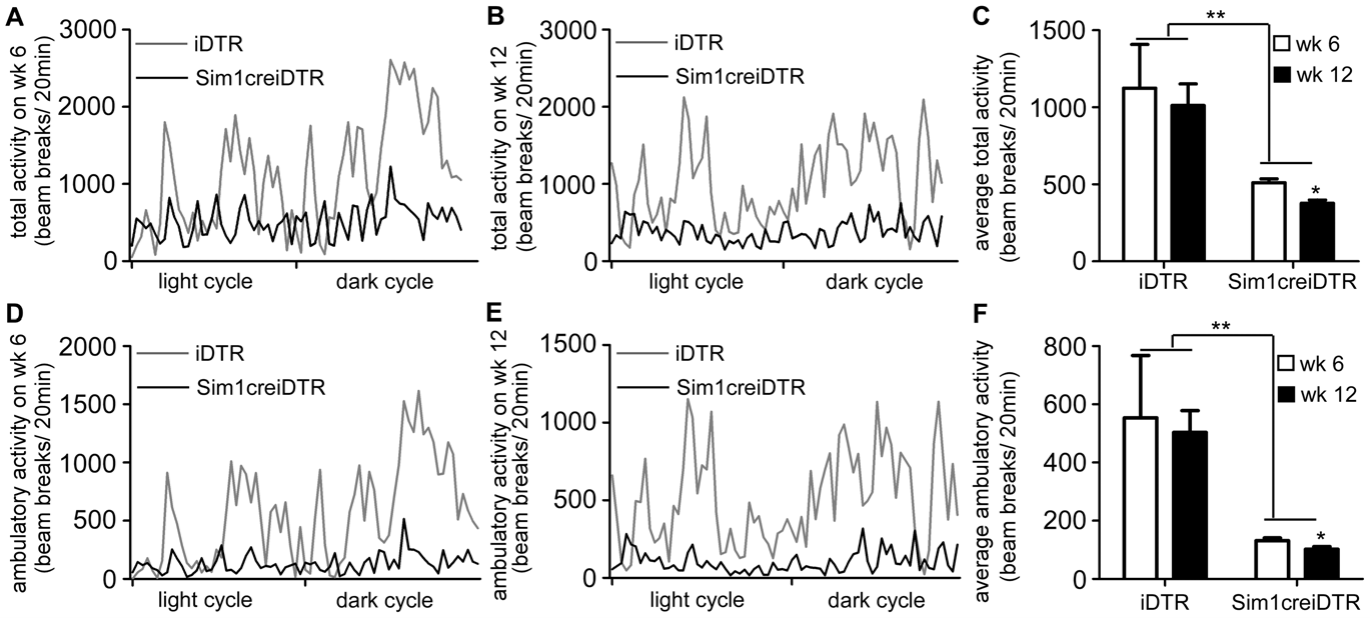
Ablation of Sim1 neurons reduces activity. Total and ambulatory activity of iDTR mice (gray line) and Sim1creiDTR mice (black line) was measured using metabolic cages at wk6 (A, D) and wk 12 (B, E). Average total (C) and ambulatory (F) activity at wk 6 and wk 12 (n = 4 for each group, *p<0.05; **p<0.01). Error bars indicate SEM for all figures. Means for each condition were compared using repeated measures two-way ANOVA with Bonferroni post-test. Total activity is any movement producing a beam break in the horizontal plane and ambulatory activity is any movement producing sequential horizontal beam breaks of different beams.

**Figure 5:**
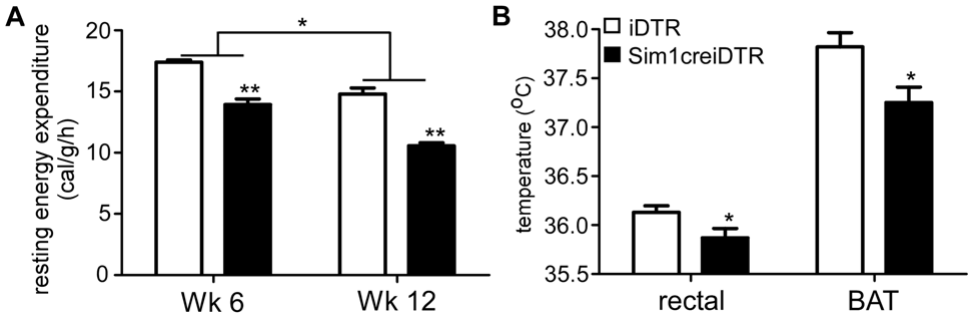
Ablation of Sim1 neurons reduces resting energy expenditure, body and BAT temperature. Resting energy expenditure for individual mice was calculated as described in the results (A, n = 4 for each group, *p<0.05, **p<0.01). (B) Rectal temperature and BAT temperature were measured (n = 7 for each group, *p<0.05). Error bars indicate SEM for all figures. Means for each condition were compared using repeated measures two-way ANOVA with Bonferroni post-test (A) and a two-tailed *t*-test, with Welch's correction (B).

*Sim1* ^+/−^ mice and conditional postnatal *Sim1^−/−^* mice have normal energy expenditure measured in the pre-obese state and are hyperphagic and develop late onset obesity after age 6 weeks. Yet bilateral PVH ablation causes reduced energy expenditure. Furthermore, pharmacologic studies show that injection of melanocortin agonists, melanocortin antagonists and NPY modulate energy expenditure when injected directly into rodent PVH [11]. To examine the participation of Sim1 neurons in energy expenditure regulation, we performed metabolic studies after DT injection and Sim1 neuron ablation but prior to the onset of obesity (wk 6 of the protocol). We found that in the pre-obese state in Sim1creiDTR mice, VO_2_, VCO_2_, and metabolic rate were decreased by 19.5%, 16.3%, 17.4% (Fig. 3A, B, D, p<0.05, df = 3) during the dark cycle, and decreased to a lesser extent during the light cycle (by 17.9%, 10.8% and 15% respectively). In the obese state, VO_2_, VCO_2_, and metabolic rate in Sim1creiDTR mice were reduced by 36.5%, 35.9%, 36.4% respectively during the dark cycle and 31.6%, 29.2%, 31.1% during the light cycle (Fig. 3E, F, H, p<0.05, df = 3). Respiratory quotient was not significantly changed in either period (Fig. 3C, G; p>0.05). Curves of total and ambulatory activity indicate that Sim1creiDTR mice are less active during both the dark cycle and the light cycle on both wk 6 (Fig. 4A, D) and wk 12 (Fig. 4B, E). Comparison between iDTR and Sim1creiDTR mice reveals that the total (p<0.01, df = 3) and ambulatory (p<0.01, df = 3) activity of Sim1creiDTR mice was significantly reduced. Only Sim1creiDTR mice exhibited reduced total and ambulatory activity with increasing age (Fig. 4C, F, p<0.05, df = 5). To assess whether decreased energy expenditure is attributable to decreased activity or altered regulation of basal metabolic rate, we calculated resting energy expenditure for each group using three different methods. We used the average of all values corresponding to ambulatory activity less than or equal to 6 [36] (shown in Fig. 5), the average of the five lowest values during a 24 h period [37], or the average of three lowest values during the light cycle [38]. Similar results were obtained from the 3 different analyses. Resting energy expenditure of Sim1creiDTR mice was significantly reduced by 21.4% in the pre-obese state and 28.7% in the obese state (Fig. 5A, p<0.01, df = 1), compared to iDTR mice. The levels of both groups on Wk 12 were lower than Wk 6 (Fig. 5A, p<0.05, df = 1). Resting VO_2_ of Sim1creiDTR mice was significantly reduced by 18.6% in the pre-obese state and 29.4% in the obese state (data not shown). Resting VCO_2_ displayed the same trend (11.1% decrease in the pre-obese state, and 26.0% decrease in the obese state, data not shown). Rectal and BAT temperature were also measured (Fig. 5B). We found that Sim1creiDTR mice had lower rectal and BAT temperatures than iDTR mice (rectal tm 35.87±0.10°C, vs. 36.13±0.13°C, p<0.05, df = 5), (BAT tm 37.25±0.16°C vs. 37.82±0.14°C, p<0.05, df = 11). mRNA expression of UCP1 in brown adipose tissue was slightly but significantly decreased in sim1creiDTR mice (0.91±0.03) compared to iDTR mice (1.00±0.01) (p<0.05, df = 18).

### SIM1 neuron ablation alters hypothalamic neuropeptide expression

**Figure 6:**
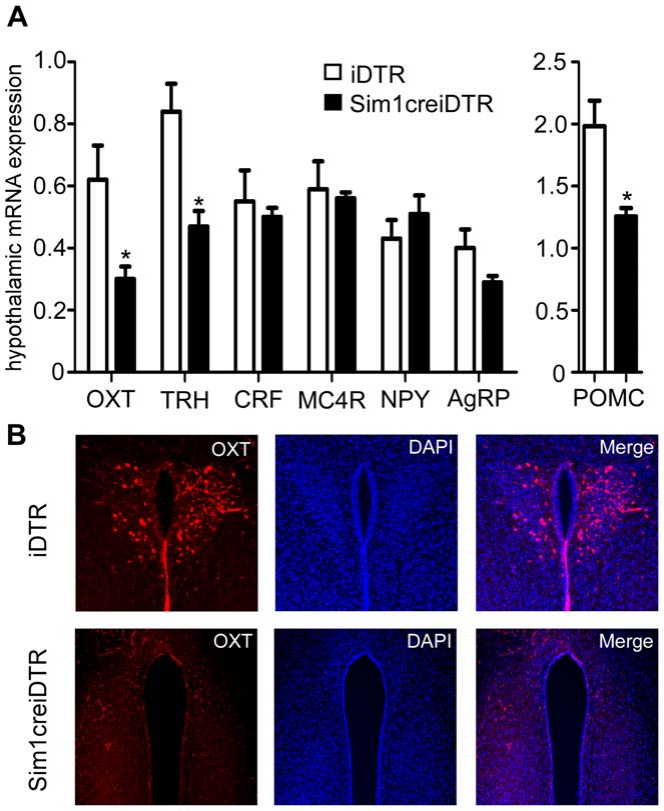
Ablation of Sim1 neurons decreases hypothalamic expression of *OXT* and *TRH*. (A) mRNA expression of obesity-related genes in the hypothalamus (n = 4 for each group, *p<0.05). (B) Immunofluorescence staining of OXT in iDTR versus Sim1creiDTR mice. Error bars indicate SEM. Means for each condition were compared by a two-tailed *t*-test, with Welch's correction.

We reported previously that Sim1 deficiency resulted in reduced oxytocin expression and impaired melanocortin-mediated anorexia as well as PVH neuron activation. Here, we investigated how Sim1 neuron ablation affected hypothalamic neuropeptide expression. In mice with Sim1 neuron ablation, we observed a decrease in *oxytocin* (*OXT*) and *thyrotropin releasing hormone* (*TRH*) mRNA levels by 51.0% and 44.7% respectively (Fig. 6A, p<0.05, df = 5 for both) relative to controls, as well as a decrease in *pro-opiomelanocortin (POMC)* by 36.7% (Fig. 6A, p<0.05, df = 5). These decreases were similar in magnitude to that observed for *Sim1* mRNA (51.8%, Fig. 2C). Interestingly, in these mice, no change in corticotropin releasing factor (*CRF*) or *MC4R* expression was observed (Fig. 6A; p>0.05). *NPY* and *AgRP* were not significantly changed. Immunofluorescence staining revealed a normal fluorescence pattern of OXT neurons in the PVH in iDTR mice (Fig. 6B, upper panels) but not in Sim1creiDTR mice treated with DT (Fig. 6B lower panels). A similar pattern was seen for SON staining (data not shown).

## Discussion

Sim1 deficiency is associated with hyperphagic obesity and normal energy expenditure [22]–[24]. Previous work has shown that reactivation of MC4Rs (in MC4R null mice) in Sim1 neurons normalized food intake with no effect on energy expenditure [20]. This result is complicated by the observation that Sim1 is expressed in neurons that are thought to regulate both feeding and energy expenditure in the PVH. The current studies were undertaken to clarify the role of Sim1 neurons in both feeding and energy expenditure regulation. Here we report diphtheria toxin mediated neuron specific ablation of Sim1 neurons in adult mice induces rapid onset hyperphagia and obesity with reduced energy expenditure. It is notable that the degree of obesity observed in these mice was inversely correlated with hypothalamic *Sim1* expression such that mice with lower hypothalamic *Sim1* expression were more obese. To avoid concerns about normalization of energy expenditure to total or lean mass, energy expenditure was measured prior to the onset of obesity. The reduction in total energy expenditure was confounded by a significant reduction in activity. Garland and colleagues showed that locomotor activity has a profound influence on total energy expenditure [39]. To address the question of whether reduced energy expenditure is merely due to a reduction in activity, we calculated resting energy expenditure using various methods including one based on energy expenditure during periods of inactivity. These analyses revealed resting energy expenditure to be significantly reduced in pre-obese mice with ablated Sim1 neurons suggesting a role for Sim1 neurons in thermogenesis. To examine this possibility, we measured body temperature, BAT temperature and BAT UCP1 expression and found significant reductions in all of these parameters in mice with ablated Sim1 neurons. It is interesting that the increase in feeding efficiency was transient. The mechanisms underlying this are not clear but may be related to homeostatic mechanisms in response to increased body weight. The finding that CRF and MC4R expression is unchanged in mice with ablated Sim1 neurons is not surprising since *CRF* and *MC4R* are both widely expressed in whole hypothalamus, and not limited to Sim1 positive neurons. However the finding that POMC expression is reduced differs from Sim1 deficient mice where POMC expression is increased. The mechanisms underlying this difference are not clear and additional mechanisms are likely present in this model where the majority of PVH neurons that express MC4R are lost whereas in the Sim1 deficient mouse models, there is decreased expression of MC4R in the PVH and resistance to MTII. Moreover, POMC deficiency itself may contribute to the obesity found in our model.

The majority of the experiments were performed in female mice. The overall weight phenotype was found to be similar but less severe in male mice. The underlying mechanisms of this sex difference are unclear. We did not examine the estrous cycle or estradiol levels in our mouse model but these could form the basis of future studies. Studies of animal models of obesity suggest that sex differences are common and related to estrogen effects [40], [41]. A common theme appears to be that body weight regulation in males is more dependent on modulation of food intake whereas weight regulation in females is more dependent on modulation of energy expenditure [42].

The specific Sim1 neurons modulating energy expenditure are unknown. Sim1 is expressed in the PVH, SON, medial amygdala and NLOT. Only the PVH has been previously implicated in energy expenditure regulation whereas both the PVH and the posterodorsal part of the medial amygdala (MePD) [43]–[46] have been implicated in feeding regulation. The relative degree of neuron ablation in our model was small in the MePD (34%) relative to the PVH (88%) suggesting a greater role for the PVH. Taken together with previous studies our results suggest that Sim1 neurons in the PVH and possibly the MePD are likely to regulate feeding while Sim1 neurons in the PVH are likely to regulate energy expenditure.

Sim1 has been shown to be critical for the development of PVH neurons [21], [47], [48]. *Sim1^−/−^* mice exhibit a failure of differentiation and migration of PVH neurons and die perinatally [21], [47]. On the other hand, *Sim1^+/−^* and conditional postnatal *Sim1^−/−^* mice have normal PVH cellularity, normal PVH projections and these neurons respond normally to stimuli such as hypertonic saline [23], [24]. These neurons fail to respond normally to melanocortin agonist activation and the mice are resistant to melanocortin-mediated anorexia but have normal melanocortin-mediated induction of energy expenditure [23]. These results suggested that the transcription factor Sim1 serves a postnatal physiologic function in feeding regulation but not in energy expenditure regulation. We showed that *MC4R* expression and *OXT* expression are reduced in the PVH of postnatal Sim1 deficient mice [24], [49]. This taken together with the fact that Sim1 deficient mice have normal energy expenditure while Sim1 neuron ablated mice have a reduced energy expenditure suggests that the transcription factor Sim1 may not be necessary in adult mice for regulating energy expenditure. By contrast, the current study suggests Sim1 neurons have a critical role in energy expenditure regulation. Although this work demonstrates a role for Sim1 neurons in regulating energy expenditure, it does not identify the PVH as the site where Sim1 neurons regulate energy expenditure. However, when considered in the light of previous work, it is unlikely that the medial amygdala, SON or NLOT are involved in energy expenditure regulation. The neuron subsets within the PVH that regulate energy expenditure are not known and our results do not address the question of neuron subtypes. Previous evidence points to TRH and OXT neurons as possibilities in this regard. TRH neurons in the PVH that project to regions regulating thermogenesis may be partly responsible for energy expenditure regulation by the PVH. TRH neurons in the anterior parvocellular PVH receive a robust innervation from NPY/AgRP and α-melanocyte stimulating hormone/cocaine-and amphetamine-regulated transcript neurons in the arcuate [50], [51]. These are non-hypophysiotropic as they do not project to the median eminence and are not regulated by thyroid hormone. These apPVH TRH neurons project to both the POA and the DMH [15]. Injection of TRH into the POA inhibits heat sensitive neurons and activates cold sensitive neurons resulting in increased body temperature through increased thermogenesis and peripheral vasoconstriction [15], [52]. In addition, injection of TRH into the DMH increases rectal temperature and BAT temperature even more potently than injection into the POA [53]. Regulation of PVH TRH neurons by leptin may be one mechanism mediating reduced thermogenesis in leptin deficient states and diet-induced thermogenesis.

Other PVH neuron subtypes that may contribute to energy expenditure regulation are OXT neurons. PRV injections into BAT infect OXT and cocaine-and-amphetamine related transcript neurons in the PVH [54] with rare infection of CRF PVH neurons. The PVH (specifically the dorsal parvocellular and ventromedial parvocellular divisions) is a major source of descending projections to sympathetic preganglionic neurons of the IML of the spinal cord [7]. OXT neurons provide the largest contribution to this projection [6] and are the greatest number of PVH neurons infected after PRV injections into BAT [54]. Thermogenesis is impaired in OXT and OXT receptor deficient mice [55], [56] and *OXT^−/−^* mice have reduced sympathetic tone [57]. Based on the above evidence, OXT neurons in the PVH are well positioned to mediate changes in EE.

In summary, our results suggest that Sim1 neurons are necessary for normal energy expenditure regulation and thermogenesis. Further experiments are needed to determine if the relevant Sim1 neurons that regulate energy expenditure are in the PVH and their specific identity and projections.

## Materials and Methods

### Ethics Statement

All procedures were carried out in accordance with the National Institutes of Health Guidelines on the Care and Use of Animals and approved by the Children's Hospital of Philadelphia Institutional Animal Care and Use Committee (Protocol #2009-10-895).

### Generation of Sim1creiDTR mice and genotyping

Sim1cre mice were obtained from Dr. Joel Elmquist and were previously characterized [20]. Homozygous iDTR mice were obtained from JAX (stock number 007900, The Jackson Laboratory, Bar Harbor, ME). Sim1cre mice were bred with homozygous iDTR mice. Animals were genotyped by multiplex PCR with the following primers: CACGACCGGCAAACGGACAGAA, TGGGATTAGCGTGTTTCAACTGAGC, TTTTGGTTTTGGATGAGTCTGTGGAG. Mice with a 250 bp band express cre while mice with a 500 bp band do not express cre.

### Experimental schedule

6-week-old females (7 Sim1creiDTR and 7 iDTR mice), were housed individually until death. This age was set as wk 1 in the protocol (Fig. 1D). Food intake and body weight were measured weekly. At Wk5, mice underwent intracerebroventricular (ICV) cannulation in the lateral ventricle and injection of DT. Metabolic studies were run at Wk6 and Wk12 to measure energy expenditure. Temperature, BAT temperature and body composition were measured before the mice were sacrificed.

### ICV cannulation and injection of diphtheria toxin (DT)

ICV cannulation and injection were performed as previously described with minor modifications described below [49]. Briefly, mice were anesthetized with isoflurane (2%, 2L O_2_/min). Using a stereotaxic apparatus (David Kopf Instruments, Tujunga, CA) a guide cannula (Plastic One Inc., Roanoke, VA) was affixed to the skull to extend 0.2 mm caudal to bregma, 1.0 mm lateral to midline, and 2.1 mm below the surface of the skull. Mice were ICV injected with 2.5 ng DT in 2.0 µl artificial CSF (aCSF). After delivery of DT, the internal cannula was left in place for 30 seconds to prevent reflux, and then slowly withdrawn.

### Feeding, and metabolic studies

All mice were fed a chow diet (PicoLab Mouse Diet 20 from Labdiet, Elkridge, MD) containing 3.75 kcal/g. Body weight and food intake were measured as previously described [24] (n = 7 for each group). At wk 6 and wk 12 of the study energy expenditure was measured using the Comprehensive Laboratory Animal Monitoring System (Columbia Instruments, Columbus, OH) (n = 4 for each group). The study was run for 3 consecutive days. O_2_ consumption, CO_2_ production, respiratory quotient (RQ) and metabolic rate (metabolic rate = (3.815+1.232×RQ)×VO_2_) in the light and dark cycle were calculated separately. Body composition was analyzed using NMR (Echo Medical Systems, Houston, TX) as described by Ahima and colleagues [58] (n = 4 for each group). Bilateral epigonadal fat was collected and weighed after the mice were sacrificed (n = 7 for each group). Rectal temperature was measured using the YSI 4600 Precision Thermometer (Fisher Scientific, Pittsburgh, PA) and brown adipose tissue temperature was measured using an implantable electronic transponder placed in the interscapular brown adipose tissue depot (BioMedic Data Systems, Seaford, DE) (n = 7 for each group).

### Immunohistochemistry

Preparation of brain samples and immunofluorescence staining were performed as previously described with minor modifications described below [49]. Briefly, Sim1creiDTR (n = 3) and iDTR mice (n = 3) were anesthetized with isoflurane and transcardially perfused with heparinized 0.9% saline, followed by 4% paraformaldehyde (PFA). The brain was removed and sectioned coronally at 30 µm using a sliding microtome (Leica SM 2000R, Buffalo Grove, IL). Sections containing PVH were blocked with 3% goat serum then incubated overnight at 4°C with mouse anti-oxytocin antibody (MAB5296, Millipore Corp., Billerica, MA) diluted 1∶5000, or rabbit anti- SIM1 antibody (AB4144, Millipore Corp., Billerica, MA) diluted 1∶1000 in 3% goat serum. Sections were then washed, incubated for 2 hours at room temperature with CY3 goat anti-mouse secondary antibody (115-165-166 Jackson ImmunoResearch Laboratories, West Grove, PA) diluted 1∶400 or CY3 goat anti-rabbit secondary antibody (111-165-003 Jackson ImmunoResearch Laboratories, West Grove, PA). Sections were mounted onto slides with vectashield mounting medium with DAPI (H-1200, Vector Laboratories, Burlingame, CA). Images containing PVH were captured using DAPI and CY3 channels using an Olympus BX61 microscope (Center Valley, PA) using Cytovision software (Applied Imaging Corp., San Jose, CA). Cell counting was performed using ImageJ software. The contrast of images was adjusted and the area where the specific nucleus is located was defined as AOI (area of interest). The particles in the AOI were counted by setting the same threshold for both groups.

### Quantitative RT-PCR

qPCR was performed as previously described with minor modifications described below [49]. Briefly, Hypothalami were dissected from fresh brains using a mouse brain block (David Kopf instruments, Tujunga, CA) (n = 4 for each group). Brown fat tissue was also collected (N = 7 for iDTR and N = 6 for Sim1creiDTR mice). Total RNA was extracted using Tripure reagent (Roche Applied Science, Indianapolis, IN). cDNA was synthesized as previously described [24]. Duplex qPCR was performed using an ABI step-one plus real-time PCR system (Applied Biosystems, Foster City, CA) and Taqman assays for the following genes with beta-actin (Cat. No. 4352341E, Applied Biosystems, Foster City, CA ) or GAPDH (Mm99999915-g1)as the control (the data shown is using actin as a control. Each qPCR experiment was repeated using GAPDH as the control, the results were similar). The following probes were used: Sim1 (Mm00441390_m1), OXT (Mm00726655_s1), TRH (Mm01182425_g1), CRF (Mm01293920_s1), MC4R (Mm00457483_s1), NPY (Mm03048253_m1), AgRP (Mm00475829_g1), POMC (Mm00435874_m1), UCP1(Mm01244861_m1).

### Data analysis

Data was analyzed using Prism Software (GraphPad Software, San Diego, CA). All values were presented as Mean ± SEM. Means were compared using a two-tailed t-test, with Welch's correction or repeated measures two-way ANOVA with Bonferroni post-tests. Differences were considered statistically significant if p<0.05 (*) and p<0.01 (**).
